# On Publishing Epidemic Constitutions

**Published:** 1802-03

**Authors:** 


### *To the Editors of the Medical and Physical Journal.*

GENTLEMEN,

IF you think the following brief Obfervations on the utility of publifhing Accounts of Epidemic Difeafes, worth a place in your valuable Journal, the infertion of them will oblige a conftant reader, and may perhaps be a means of increafing the beneficial effects that have already accrued from its extenfive circulation.

Among the numerous defiderata in medical fcience, there is fcarcely any of greater importance, or which is likely to be attended with happier effects, than an accurate account of epidemic mic difeafes, delivered by an attentive obferver, who not fuffering himfelf to be fwallowed up in theoretical difquifitions, will be content to relate plain facts, to lay before the public the peculiarity of fymptoms and diverfities of appearance which he has feen, and to deliver that mode of treatment which he has found moft fuccefsful. The moft valuable labours of the Coan Sage that we poffefs, are hiftories of this kind; and fimilar ones are regarded as the moft precious legacies of our excellent Sydenham, and have procured for him the title of the Englifh Hippocrates. But in the prefent day, when men of learning are fo attached to, and dazzled with the allurements of theory, that every thing which does not immediately coincide with their adopted opinion is haftily rejected, this fubject, which demands a cool enquiry and impartial judgement, has been univerfally (I had almoft faid fhamefully) neglected; and fince the days of Huxham, Sydenham, and the older phyficians, there has fcarce appeared in this country, any work on Epidemic difeafes. It may probably be anfwered, that no epidemic of importance has prevailed; but a fmall Tract, publifhed fome time fince by Dr. Pearfon of Birmingham, and a very recent one by Mr. White of Bath, prove that fuch affections have exifted, and induced me to attribute this filence of our brethen to inattention.

I remember to have feen fome time ago in this Journal, a few excellent obfervations on Hydrocephalus Internus by the latter gentleman, and have now derived great pleafure from the perufal of his Account of the Bilious Fever which has prevailed at Bath for fome years back. It adds, I think, much to the author's merit, that he has not indulged in any fpeculative theory, but contented himfelf with a plain narrative of facts, told with much perfpicuity and judgement, and enriched with much collateral evidence from other writers. The work, however, would in my opinion have fuffered no lofs, had his obfervations on the nature of epidemics and fome of the cafes been curtailed, Neverthelefs it is well worth the attention of any medical practitioner, and it prefents an excellent plan for any that may be induced to undertake fo meritorious a work.

Should thefe obfervations chance to arreft the attention of any of your readers, I hope they will deem them worthy of notice; having at the fame time candour enough (which I muft alfo requeft of you) to attribute the poor form in which they appear, to its being almoft the firft time that any thing has appeared in public, from

Your's, &c.

PHILO-MEDICUS LONDINENSIS.

